# Three-Dimensional Printable Color-Modulation and Shape-Programmable Structures: An Encryption Key for Image Recognition Electronic Locks

**DOI:** 10.34133/research.0666

**Published:** 2025-04-25

**Authors:** Beibei Du, Xiayu Zhang, Teng Wang, Yunfei He, Mingyao Shen, Tao Yu

**Affiliations:** ^1^ Frontiers Science Center for Flexible Electronics (FSCFE) and Xi’an Institute of Flexible Electronics (IFE), Northwestern Polytechnical University (NPU), Xi’an 710072, China.; ^2^ School of Automation, Northwestern Polytechnical University (NPU), Xi’an 710072, China.

## Abstract

Stimuli-responsive materials have shown promising applications in the areas of sensing, bioimaging, information encryption, and bioinspired camouflage. In particular, multi-stimuli-responsive materials represent a hot topic due to their modulated properties under multiple stimuli. Herein, we successfully developed multi-stimuli-responsive inks and a series of complex multi-stimuli-responsive 3-dimensional (3D) structures were fabricated via digital light processing 3D-printing technology. Notably, these complex 3D structures show shape memory, fast-response photochromic and thermochromic behavior, and excellent repeatability due to the combination of photochromic molecules (4-(2,2-bis(4-fluorophenyl)vinyl) benzyl methacrylate) and thermochromic pigments. Furthermore, a programmable encrypted box that changes colors and morphology by controlling temperature and ultraviolet irradiation was designed and printed, and this encrypted box exhibits strong security using OpenCV-based image recognition technology. This strategy provides a promising approach for the design of multi-stimuli-responsive materials and complex encryption systems in the future.

## Introduction

The ability to morph or change color is essential for some plants and animals, such as the on/off switch of Venus flytraps, phototropism in sunflowers, and color change of chameleons according to surroundings. With this inspiration, stimuli-responsive polymers that exhibit special functions in response to external conditions have attracted increasing attention [1–9]. Accordingly, the construction of programmable 3-dimensional (3D) structures, which exhibit special functions in response to external conditions, has become a hotspot in recent years. Shape-memory polymers (SMPs) are one of the most extensively used stimuli-responsive materials, which depend on an elastic network and a temporary network to realize deformation with temperature change over time [8,10–13]. In previous studies, programmable 3D structures based on SMPs can endow polymers the capability to respond to environmental stimuli [14–17], including heat [18], moisture [19], magnetic field [1], or electricity, with changing time. Complex construction and design of SMP-based functional polymers has been a long-pursued goal.

Important efforts have been devoted to adding functions into SMP-based functional polymers in recent years. However, only a few papers report the synthesis of multi-stimuli-responsive polymers based on conventional methods (for example, doping or the postprocessing method) [20,21], which has the disadvantages of solvent-induced mechanism; unquantified, nonhomogeneous functional partials; and poor solvent and thermal resistances. Thus, combining photochromic materials with SMP materials could bring more complex structures and functions by just taking advantage of stimuli such as heat or light [22–24].

Photochromism is a typical photoresponsive phenomenon that displays reversible obvious color transformation under ultraviolet (UV) light stimulation [25]. Compared with other kinds of stimuli, UV light shows the great advantages of easy access, nondirect contact, and high precision [26]. The UV-light-induced photochromism phenomenon can be realized using dithienylethene derivatives [27–29], azobenzene derivatives [30–33], spiropyran derivatives [34,35], fulgide derivatives [36,37], and other systems. Triphenylethylene derivatives are a series of novel photochromic systems with simple chemical structures and decent photoresponsive properties [38,39].

**Fig. 1.**
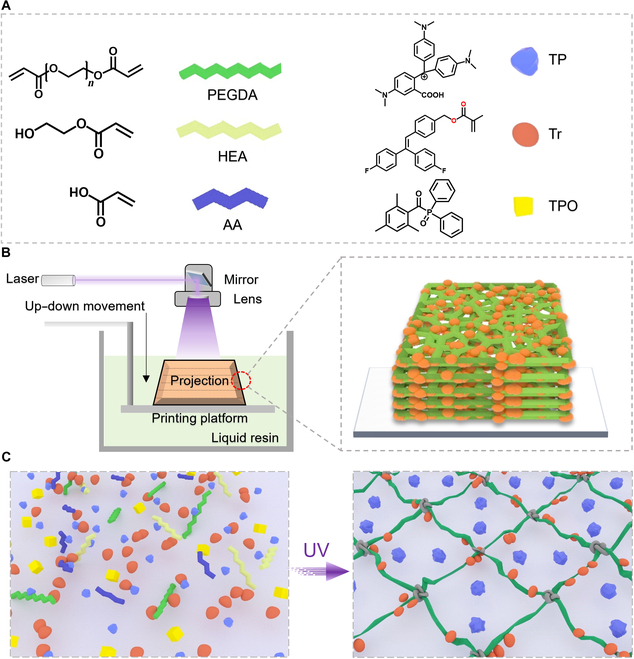
Construction of multi-stimuli-responsive 3-dimensional (3D)-printable materials. (A) Detailed chemical structures of 4-(2,2-bis(4-fluorophenyl)vinyl) benzyl methacrylate (TrPEF_2_-MA, Tr), thermochromic pigment (TP), diphenyl(2,4,6-trimethylbenzoyl) phosphine oxide (TPO), acrylic acid (AA), 2-hydroxyethyl acrylate (HEA), and α,ω-diacryloyl poly(ethylene glycol) (PEGDA) that are used to prepare the Tr-TP shape-memory polymer (SMP) precursor solution. (B) Schematic diagram of the layer-by-layer stacking digital light processing (DLP)-based 3D-printing process. (C) Illustrations of the photopolymerization process during DLP-based 3D printing. Left: Tr-TP SMP precursor solution before 3D printing. Right: Tr-TP SMP network structure after 3D printing. UV, ultraviolet.

Herein, we prepared multi-stimuli-responsive 3D-printable resins by a combination of photochromism and thermochromism with SMP materials that show synchronized abundant color-modulation and shape-changing behaviors. The behaviors were further programmed and successfully applied to information security. We prepared a photochromic molecule (4-(2,2-bis(4-fluorophenyl)vinyl) benzyl methacrylate [TrPEF_2_-MA, Tr]) based on previous research [40] as a candidate for preparing UV-curable photochromic resins and introduced thermochromic pigments (TPs) as other reversible color-changing materials that respond to temperature. The TP relies on an electron gain and loss mechanism to switch from color to white at elevated temperatures. We selected 3 TP materials, which are green TP materials (TP1), red TP materials (TP2), and blue TP materials (TP3). TP1 was an example, and 2-hydroxyethyl acrylate (HEA), acrylic acid (AA), and α,ω-diacryloyl poly(ethylene glycol) (PEGDA) are preferred matrixes for preparing SMPs due to their biocompatibility [41], easy dyeing [42], and good shape-memory function [43,44]. By mixing TrPEF_2_-MA and TP to the SMP matrix and using diphenyl(2,4,6-trimethylbenzoyl) phosphine oxide (TPO) as a photoinitiator, UV-curable liquid resins were successfully prepared (Fig. 1A). As shown in Fig. 1B, by using layer-by-layer stacking digital light processing (DLP)-based 3D printing, the resin can be prepared into highly complex 3D structures with high resolution up to ca. 20 μm on the vertical side. As illustrated in Fig. 1C, the solidified 3D structures were macromolecular-based networks, which were composed of cross-linked monofunctional monomers, grafted TrPEF_2_-MA functional groups, and uniformly doped TP. The structures exhibited conspicuous shape-memory behavior and thermochromic and photochromic ability, which were highly repeatable. Not only that, we used the 3D-printed deformed and color-changing structure in OpenCV-based image recognition, preparing a unique decryption key that is difficult to copy. Such a 3D structure with outstanding mechanical properties, good shape-memory ability, and multiple discolorations has promising applications in information protection and display, flexible electronics, and biomedical fields.

## Results and Discussion

TrPEF_2_-MA (Tr) was synthesized according to the previous report from our group. TrPEF_2_-MA can be UV cured and has an excellent color change effect, which can vary from transparent to orange by UV irradiation of 365 nm due to the reversible ring-closure reaction of the triphenylethylene moieties, as illustrated in Fig. S1. To investigate the photochromic property of the 3D-printed structures based on TrPEF_2_-MA, 3 types of resins were printed by combining SMP and Tr/TP, which were named Tr SMP, TP SMP, and Tr-TP SMP (the corresponding compositions and contents are listed in Table 1).

**Table 1.** Materials used in the UV-curable liquid resin

Liquid resin	SMP	Tr SMP	TP SMP	Tr-TP SMP
PEGDA	1 wt.%	1 wt.%	1 wt.%	1 wt.%
AA	69 wt.%	62 wt.%	65.5 wt.%	58 wt.%
HEA	29 wt.%	25 wt.%	27.5 wt.%	25 wt.%
TPO	1 wt.%	1 wt.%	1 wt.%	1 wt.%
TrPEF_2_-MA (photochromic molecule)	ND	10 wt.%	ND	10 wt.%
TP (thermochromic pigment)	ND	ND	5 wt.%	5 wt.%

ND, no data

**Fig. 2.**
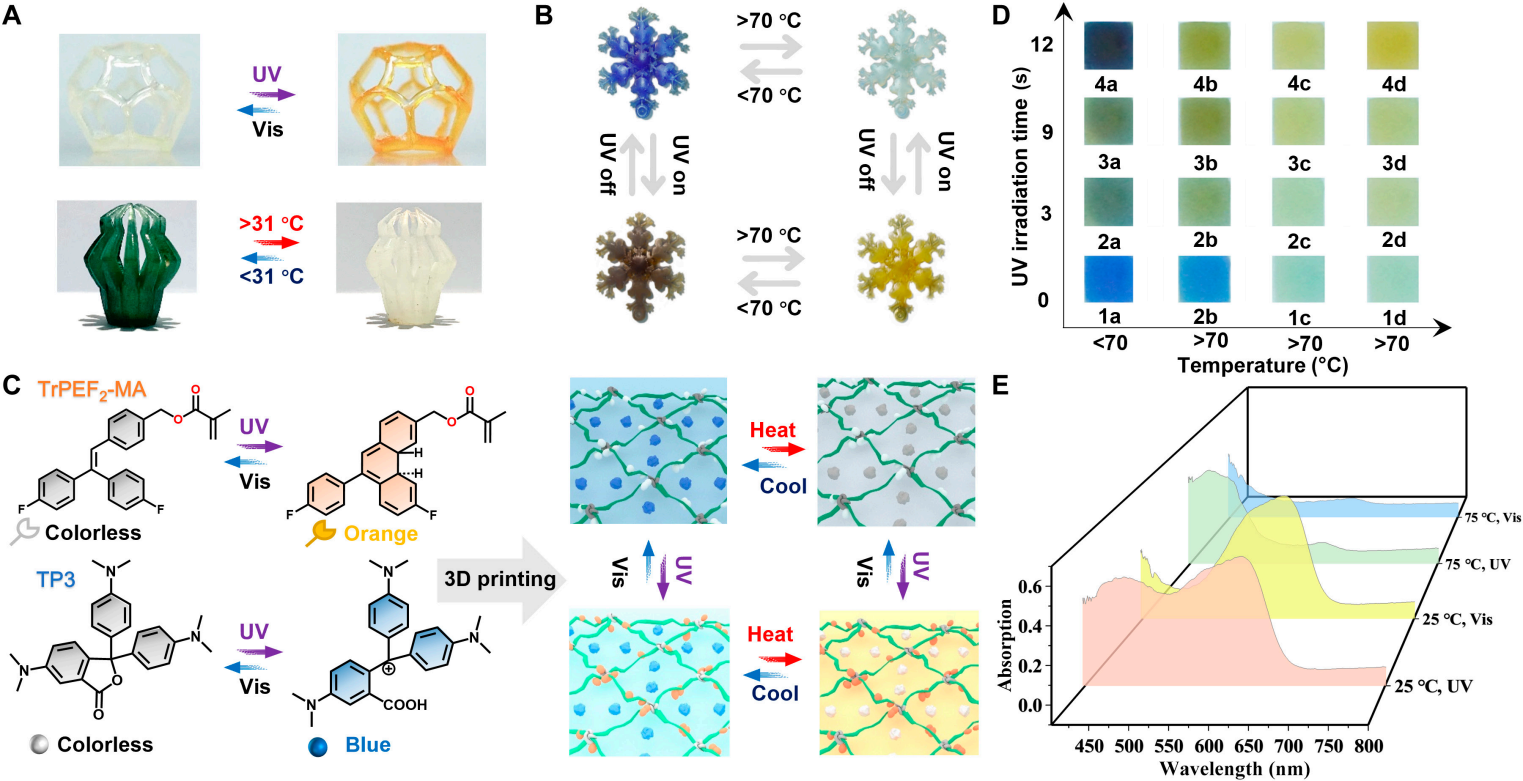
Discoloration performance and mechanism of multi-stimuli-responsive materials. (A) Three-dimensional printing structures of Tr SMP (up) and TP1 SMP (down). (B) The color change schematic of 3D printing snowflakes in different states using Tr-TP3 SMP. (C) Left: discoloration mechanism of TrPEF_2_-MA (up) and TP (down) materials. Right: chemical structure evolution of liquid resin during changes in ambient temperature and light. (D) Color change of a spline in different states. (E) UV–visible absorption spectra of a spline at different temperature and light conditions. Vis, visible.

Tr SMP containing 10 wt.% TrPEF_2_-MA could fulfill 2-color switches by manipulating the UV–visible (Vis) irradiation, whose color can repeatedly vary from transparent to yellow. Tr SMP showed excellent photochromic properties and excellent photochromic repeatability, as shown in Fig. 2A and Figs. S2 and S3, and its intense absorption band with a maximum of 470 nm was observed under UV irradiation (365 nm) within 60 s. We prepared 3 kinds of TP SMP (TP1: green, responsive temperature of 31 °C; TP2: red, responsive temperature of 50 °C; and TP3: blue, responsive temperature of 70 °C). The mechanism of thermochromic molecular structures (TP1, TP2, and TP3) is illustrated in Figs. S4 to S8. The time-dependent UV–Vis absorption spectra of a printed 3D structure with TP1 SMP were collected. The 3D structure at 35 °C exhibited a vast difference in color compared with that under 25 °C. TP1 is green below 31 °C and rapidly changes to clear when the temperature is above 31 °C. In addition, through conducting 25 photochromic and thermochromic cycles, the decent reversibility color-responsive properties of the 2 types of printed structure are demonstrated in Fig. 2A and Figs. S9 to S12. The discoloration mechanism of TrPEF_2_-MA and TP in Tr-TP SMP is shown in Fig. 2C. Ionic bonding and hydrogen bonding can be thermally reversibly broken and reformed in TrPEF_2_-MA, which endows the printed objects with more stable photoresponsive functions compared with the doped polymer. TP is added to Tr-TP SMP in the form of blending and exhibits a great discoloration effect when the temperature changes due to the electron gain and loss mechanism. A 3D snowflake, as shown in Fig. 2B, was directly printed by DLP-based 3D printing with Tr-TP SMP (composite resin containing 10 wt.% TrPEF_2_-MA and 5 wt.% TP3) and shows the richest color variations. The structure exhibited a uniform blue color under visible light at room temperature. When the temperature was gradually increased to 70 °C, the blue color progressively faded and turned white. Under UV light (365-nm) irradiation, the TrPEF_2_-MA compound in a 3D structure underwent a reversible ring-closure reaction, giving a homogeneous yellow color. Color variations could be tuned by adjusting different combinations of temperature and UV irradiation times, as shown in Fig. 2D. The detailed light absorption abilities under 4 kinds of conditions were shown in the UV–Vis absorption spectra (Fig. 2E), corresponding to the discoloration effects from blue to white, yellow, and brown. Hence, by combining the TrPEF_2_-MA and TPs with a functional monomer, the multicolor programmability of the 3D-printed structures was successfully achieved. Moreover, Tr and TP3 in doped polymethylmethacrylate (PMMA) films were also studied. The UV–Vis absorption spectra of Tr-TP3 PMMA film showed that the Tr-TP3 PMMA polymer and Tr-TP3 SMP polymer have the same photochromic and thermochromic behavior (Fig. S13).

**Fig. 3.**
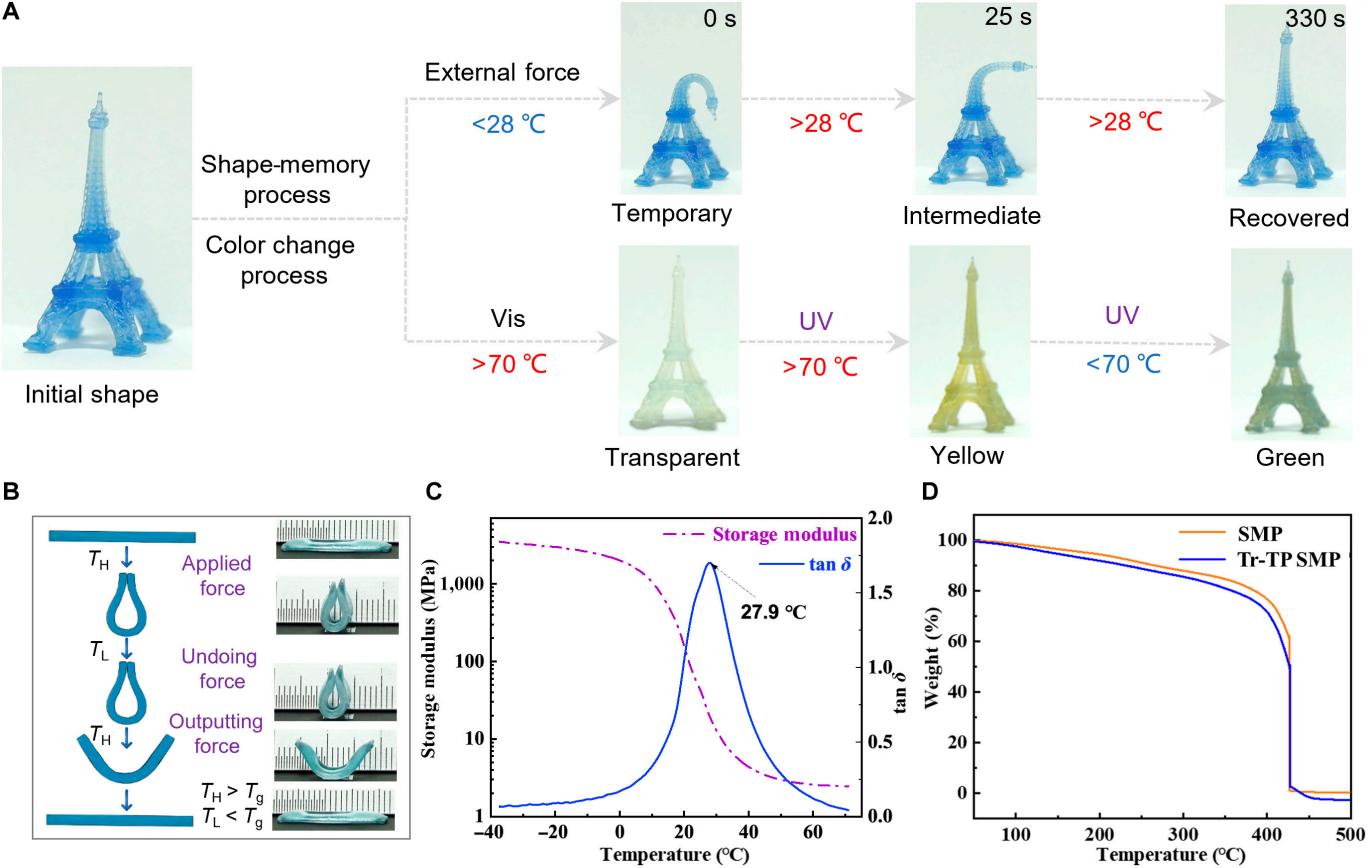
Shape-memory and thermal properties of Tr-TP SMP. (A) Shape-recovery and discoloration abilities of the printed “Eiffel Tower” model under different conditions. (B) Schematic diagram of the deformation mechanism of SMP. (C) Dynamic mechanical analysis (DMA) of the printed Tr-TP SMP resin. (D) Thermogravimetric analysis (TGA) of the printed Tr-TP SMP resin.

Furthermore, to demonstrate the thermo- and photoresponsive properties of Tr-TP SMP, we designed a printed model of the Eiffel Tower, as shown in Fig. 3A. The “Eiffel Tower” was curved by an external force and was heated above the *T*_g_ (27.9 °C) to form a temporary curved status (temporary status in Fig. 3A). With the temperature going down, the printed model could hold its curved shape (temporary shape) while undoing the external force. At this time, several transition shapes in different states can be observed with slowly increasing temperature. Finally, the printed Eiffel Tower returned to its original shape at room temperature. In addition, the printed Eiffel Tower model showed a color-changing ability in the process of deformation. Moreover, we printed a series of 3D structures such as bionic grippers, hollow balls, and bionic octopuses (Figs. S16 to S18) to verify their excellent color- and shape-responsive performances under different temperatures and light conditions.

To quantify the shape-memory property of a 3D structure using Tr-TP SMP, we printed a spline and calculated the shape-fixing ratio (*R_f_*) and shape-recovery ratio (*R_r_*) as shown in Fig. S19. The initial angle of the spline was recorded as $\alpha =180^{{}^{\circ}}$. The spline was heated to 50 °C, programmed to bend at *β*, and fixed by cooling with an ice bath, and the fixed angle γ was recorded, calculating $R_{f}=\gamma \;/\;\beta =94.17\%$. When heated up again, the spline returns to an angle δ, calculating $R_{r}=\delta \;/\;\alpha =96.67\%$, proving that printed structure with Tr-TP SMP has the advantages of high shape-memory rate, high shape-recovery rate, and excellent shape-memory performance. Its deformation mechanism is shown in Fig. 3B, which could also be summarized as constrained thermomechanic [45]. First, the spline was heated (*T*_H_) beyond the glass transition temperature (*T*_g_). An external force was applied to this spline to achieve a temporary shape. After cooling to *T*_L_ (*T*_L_ < *T*_g_), the spline could remain in the temporary shape through undoing external force. Then, the structure can be returned to its original shape again by heating to *T*_H_. To investigate the thermomechanical properties of the 3D-printed structures with Tr-TP SMP, we used dynamic mechanical analysis and thermogravimetric analysis for analysis (Fig. 3C). The results showed that the material would not decompose significantly until the material’s temperature is greater than 200 °C, much higher than the material’s *T*_g_ (27.9 °C) and discoloration temperature range, which implies high stability of Tr-TP SMP, allowing dozens of bending and color changes. The temperature-dependent storage modulus and tan *δ* of the 3D-structure-based SMP are shown in Fig. S20. According to the peak of the corresponding tan *δ* curve, the *T*_g_ values of the printed-structure-based SMP and Tr-TP SMP were 40.9 and 27.9 °C, respectively. The lower *T*_g_ of Tr-TP SMP is mainly ascribed to the addition of TrPEF_2_-MA, and TP decreases the cross-linking density of the SMP system, resulting in higher polymer chain mobility, thus lowering *T*_g_ according to previous studies [46]. These thermodynamic properties show its potential as a great shape-memory material. In addition, the mechanical tensile curves indicate that introducing Tr and TP molecules into the material has not significantly influenced its mechanical properties (Figs. S21 and S22).

**Fig. 4.**
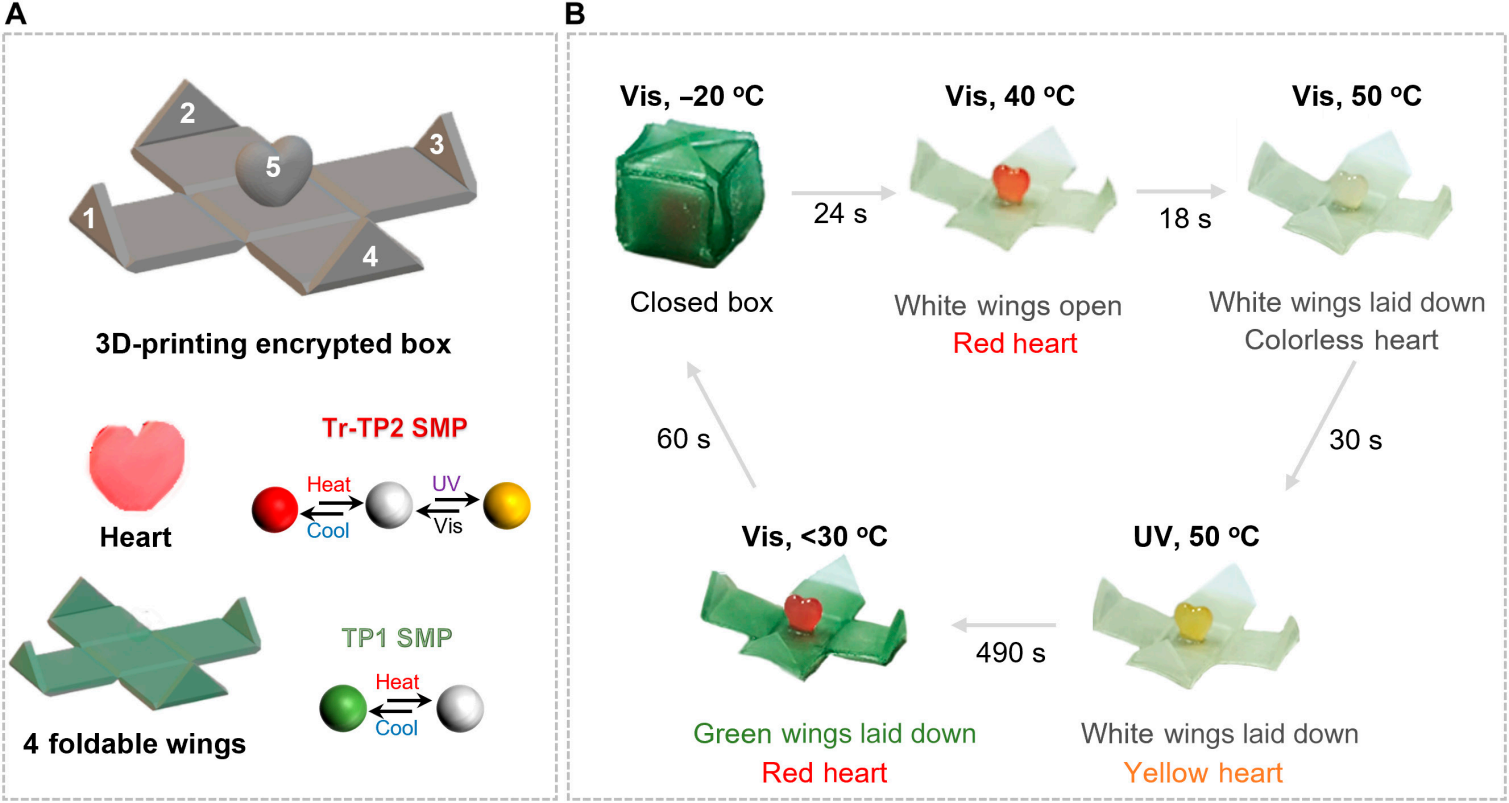
Schematic of a “heart-in-box” (HiB) 3D structure. (A) The 3D structure of the HiB model and corresponding compositions, where the heart is made of Tr-TP2 SMP and 4 foldable wings were made of TP1-SMP. Five marked points to calculate HSV values and schematic of the 3D-printed encryption box by liquid resin. (B) The programming process of the printed encryption box through shape and color change under temperature and light dual stimuli.

By further taking advantage of the thermo- and photochromic properties, a “heart-in-box” (HiB) 3D structure was designed and successfully fabricated by DLP-based 3D printing. As shown in Fig. 4A, the structure consists of a heart-shaped component and 4 foldable wings surrounding it; the materials used for this structure are TP1 SMP (foldable wings) and Tr-TP2 SMP (heart). More printing details are shown in Table S1. Under the stimulation of temperature and UV irradiation, the HiB could implement abundant color and structural changes. For example, the HiB stayed closed with an external force at −20 °C. When the temperature increased to 40 °C (above *T*_g_), due to the shape-memory effect, the 4 foldable wings in green reopened and the inner red heart was exposed. When the temperature rose to 40 °C, the wings gradually returned from green to white. If the temperature continues to increase until the critical temperature of the color change of the “heart” part is reached, the entire structure will become white. Afterward, the color of inner heart changed from colorless to orange-yellow under UV irradiation of 365 nm (temperature fixed). Subsequently, TrPEF_2_-MA reopened the ring from yellow to colorless under visible light with the temperature unchanged. Finally, as the temperature decreased below 31 °C, the box portion turned green again to finish the cycle.

**Fig. 5.**
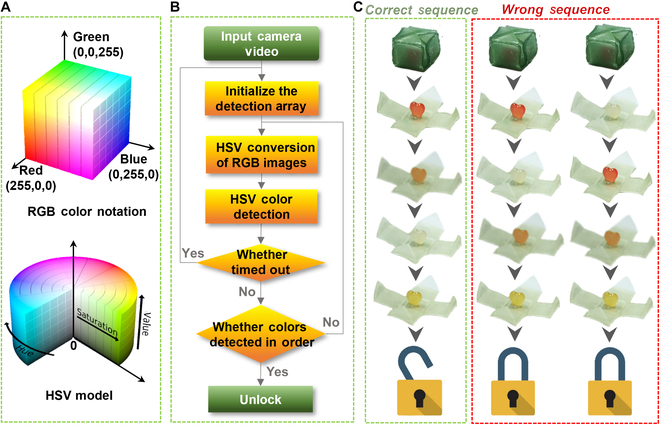
Schematic information encryption technology. (A) Conversion model of RGB to HSV. (B) The pseudocode in the image recognition process. (C) Diagram of the correct password sequence.

Based on the color and shape change performance of the above materials, an encryption method based on color image identification is proposed. The principle is mainly to use the colors and states expressed by the 5 given parts of HiB as the main components of the key as shown in Fig. 4A. Among them, the colors and status of the HiB can be recognized by the color capture program supported by OpenCV, which is compared with the HSV value in the pre-defined correct state by the system in advance. The conversion model of RGB to HSV is shown in Fig. 5A. The conversion equation is shown as follows:

x=s×coshy=s×sinhz=v (1)

where x, y, and z represent the grayscale values of the 3-color channels in the RGB image and h, s, and v represent the image hue, saturation, and brightness, respectively. The extraction process of 4 color HSV values is shown in Fig. S21. Figure 5B illustrates the algorithm steps based on computer identification systems and codes. First of all, the coordinates of the 5 parts are then converted into corresponding HSV codes, as shown in Table 2. Then, we used the 5 HSV codes obtained under a single condition as a set of cyphers and designed a randomly arranged cypher set as the key. We provide an example of a password group in Fig. 5C. The external environment was adjusted in the order of “UV off and heating between 31 and 50 °C → UV on and keeping the temperature unchanged → UV off and heating above 50 °C → UV on and keeping the temperature unchanged”. The hardware composition is shown in Fig. S23. The computer system recognizes the sequence of HSVI, HSVII, HSVIII, HSVIV, and HSVV; the signal can only be transmitted to the relay; and the electromagnetic lock is controlled to open, thus achieving the door lock opening. Otherwise, the safety lock will be closed. Interestingly, if the HiB is changed in 5 states by controlling the external temperature and light, the HSV value detected by the computer identification system has a total of 120 permutations, with only one correct order. Therefore, based on this premise, the probability of cracking the password is 1/120. Meanwhile, without restricting the changes of HiB in 5 states, there are countless combinations that significantly reduce the likelihood of password decryption and dramatically improve security. The proposed encryption method helps to overcome the issues of insecurity and easy replication in current encryption locks, thereby demonstrating the huge application prospects of 3D-printed dual-response resin in data encryption.

**Table 2.** The different HSV values corresponding to each of the 5 points of the HiB 3D structure in 5 states

Point	HSVI	HSVII	HSVIII	HSVIV	HSVV
Point 1	32 79 52 151 41 181	0 58 0 59 157 195	39 51 30 50 170 206	39 60 24 53 155 203	39 59 30 55 159 202
Point 2	32 79 52 151 41 181	0 58 0 59 157 195	39 51 30 50 170 206	39 60 24 53 155 203	39 59 30 55 159 202
Point 3	32 79 52 151 41 181	0 58 0 59 157 195	39 51 30 50 170 206	39 60 24 53 155 203	39 59 30 55 159 202
Point 4	32 79 52 151 41 181	0 58 0 59 157 195	39 51 30 50 170 206	39 60 24 53 155 203	39 59 30 55 159 202
Point 5	32 79 52 151 41 181	3 27 110 186 153 198	14 20 109 187 161 192	31 39 37 65 162 209	25 30 81 179 160 211
State	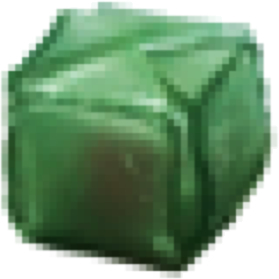	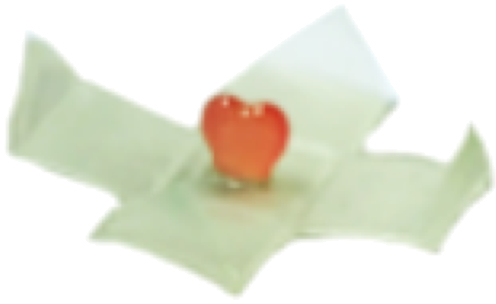	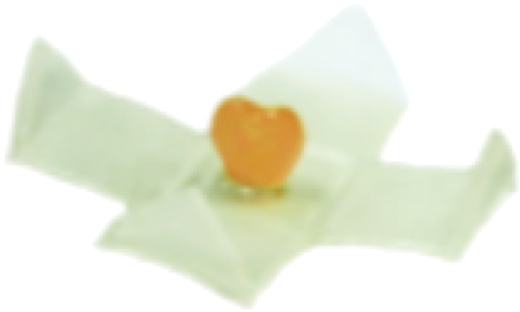	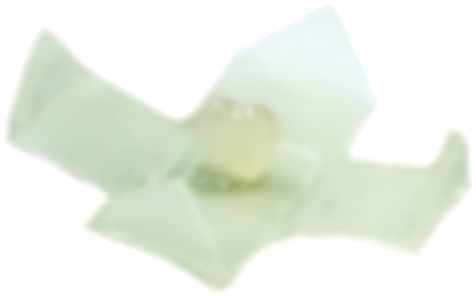	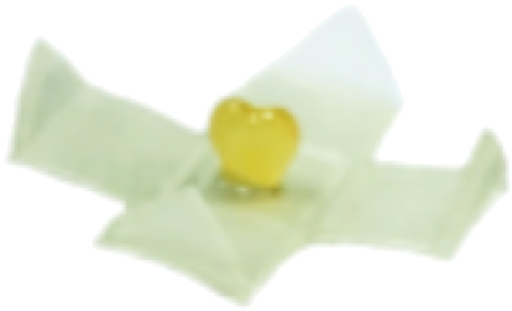

## Conclusion

In summary, a series of novel SMPs with reversible photochromic and thermochromic behaviors were successfully designed and synthesized. Remarkably, a variety of prescribed complex 3D structures with a high resolution (ca. 20 μm on the vertical side) were successfully fabricated based on the DLP 3D-printing technique. These 3D structures show striking shape–color responsiveness, multicolor (more than 16 colors) controllability, and good repeatability under light irradiation and temperature change. More importantly, a shape–color-programmable 3D structure was designed and printed, which solved the security problem of password lock via RGB–HSV conversion and the call of the OpenCV library. This work not only represents a rare example of multi-stimuli-responsive 3D-printable materials but also provides a new strategy for applications in multifunctional optical storage, soft robots, information encryption, and bioinspired camouflage.

## Materials and Methods

The materials and methods are presented in the Supplementary Materials.

## Data Availability

All relevant data that support the findings are available within this article and its Supplementary Materials and are also available from the authors upon reasonable request.

## Supplementary Materials

Supplementary 1 Supplementary Materials and Methods Figs. S1 to S23 Tables S1 and S2 Videos S1 to S4
