# Supplementary material for: Three-Dimensional Printable Color-Modulation and Shape-Programmable Structures: An Encryption Key for Image Recognition Electronic Locks
Source: Research (Wash D C). 2025 Apr 25;8:0666. doi: 10.34133/research.0666 (PMC12022503; doi:10.34133/research.0666)
Supplement: Supplementary 1 — Supplementary Materials and Methods Figs. S1 to S23 Tables S1 and S2 Videos S1 to S4 [file research.0666.f1.docx]

**Supporting Information**

3D Printable Color-modulation and Shape Programmable Structures: An Encryption Key for Image Recognition Electronic Lock

*Beibei Du^1†^, Xiayu Zhang^1†^, Teng Wang^2^,Yunfei He^1^, Mingyao Shen^1^, Tao Yu*^1^*

**Abstract:** Stimuli-responsive materials have shown promising applications in the areas of sensing, bio-imaging, information encryption, and bioinspired camouflage. In particular, multi-stimuli-responsive materials represent a hot topic due to their modulated properties under multi-stimuli. Herein, we successfully developed multi-stimuli-responsive inks and a series of complex multi-stimuli-responsive 3D structures were fabricated via digital light processing (DLP) 3D printing technology. Notably, these complex 3D structures show shape memory, fast-response photochromic and thermochromic behaviour, and excellent repeatability due to the combination of photochromic molecules (Tr) and thermochromic pigments (TP). Furthermore, a programmable encrypted box that changes colours and morphology by controlling temperature and UV irradiation was designed and printed, and this encrypted box exhibits strong security using OpenCV-based image recognition technology. This strategy provides a promising approach for the design of multi-stimuli-responsive materials and complex encryption systems in the future.

Table of Contents

[Table of Contents 2](#_Toc187320829)

[Supplementary materials and methods 3](#_Toc187320830)

[Materials 3](#_Toc187320831)

[Preparation of photo-curable liquid resin 3](#_Toc187320832)

[3D printing fabrication of resins 3](#_Toc187320833)

[Characterization 3](#_Toc187320834)

[Supplementary Figures 4](#_Toc187320835)

[Supplementary Tables 12](#_Toc187320836)

[Supplementary Movies 12](#_Toc187320837)

Supplementary materials and methods

Materials

Methyl 4-(Bromomethyl) Benzoate, Triethyl phosphite, 4,4'-Difluorobenzophenone, Potassium Tert-Butoxide (t-BuOK), Disobutylaluminium hydride (DIBAL-H), Methacryloylchloride, Triethylamine, Magnesium sulfate, n-Hexane, Ethanol, Dichloromethane (CH_2_Cl_2_), Tetrahydrofuran (THF), 2-hydroxyethyl ester (HEA), acrylic acid (AA), α,ω-Diacryloyl Poly(Ethylene Glycol) (Mn=700) (PEGDA), Diphenyl (2,4,6-trimethylbenzoyl)phosphine oxide (TPO). All reagents and solvents mentioned above were purchased from Aladdin, Titan or Adamas, and were used as received. Thermochromic microcapsules were purchased from Shenzhen Dongfangbianse Technology Co., Ltd.

Preparation of photo-curable liquid resin

The liquid resins were prepared by mixing HEA, AA, PEGDA with different weight fractions, and stirred for 1h to make the resins uniform and stable. Then, the photoinitiator (TPO) was added of 1wt.%. The thermochromic microcapsules and photochromic materials were mixed in the resin mentioned above at ratios of 5% and 10%. The liquid resins were well prepared for following 3D printing.

3D printing fabrication of resins

The 3D model to be printed was designed by software (Solid Works) and was sliced into 2D images to the required thickness using another software (BMF Slicer). Then, the designed 3D model was obtained by stacking layer by layer photocuring through digital light process (DLP) 3D printing (Nano Arch P150) with an x-y axis resolution of 50 μm. After printing, the obtained structure was washed with ethanol to remove unreacted monomers and uncured oligomers from the surface and put into UV for 5 minutes oven to finish post-curing.

Preparation method of Tr-TP3 PMMA films

At room temperature and in a dark environment, 1 g PMMA, 0.1 g Tr, and 0.05 g TP3 were dissolved in 10 mL of tetrahydrofuran solution, and ultrasonic dispersion was performed for 90 minutes. 8 mL of the above solution was poured into a polytetrafluoroethylene mold and placed in a dark room at room temperature for one night to evaporate, thereby obtaining a Tr-TP3 PMMA film.

Characterization

UV-Vis absorption spectra, photoluminescence (PL) spectra, and transmittance spectra were obtained on a UV-Vis spectrometer (Hitachi U-3900H), a Hitachi F-7100 fluorescence spectrophotometer and an Ocean Optic QE 65Pro spectrometer with Ocean Optic reflection probes R600-125F. The wavelength and intensity of UV excitation light is 365 nm and 5W flashlight. The differential scanning calorimetry (DSC) and thermal gravimetric (TG) tests were measured by differential scanning calorimetry (DSC, NETZSCH 214 Germany) and thermal gravimetric analyzer (TG, NETZSCH 209 F3, Germany), and the printed samples were kept at the heating rate of 10 °C·min^-1^ from 30 °C to 500 °C. Dynamic mechanical properties were studied by using a dynamic mechanical analysis (DMA) analyzer (DMA, NETZSCH 242E, Germany) in the tension film mode. Samples for all the compositions were trimmed to a typical dimension of 10.000 mm × 0.900 mm × 4.800 mm and tested at a frequency of 1 Hz, a “force track” of 125% and an amplitude of 10 μm. The temperature was heated up at a heating rate of 10 °C/min. Dynamic thermomechanical analysis was used to measure storage modulus (*E*′), loss factor (tan*δ*), and tensile test at different temperatures.

Supplementary Figures


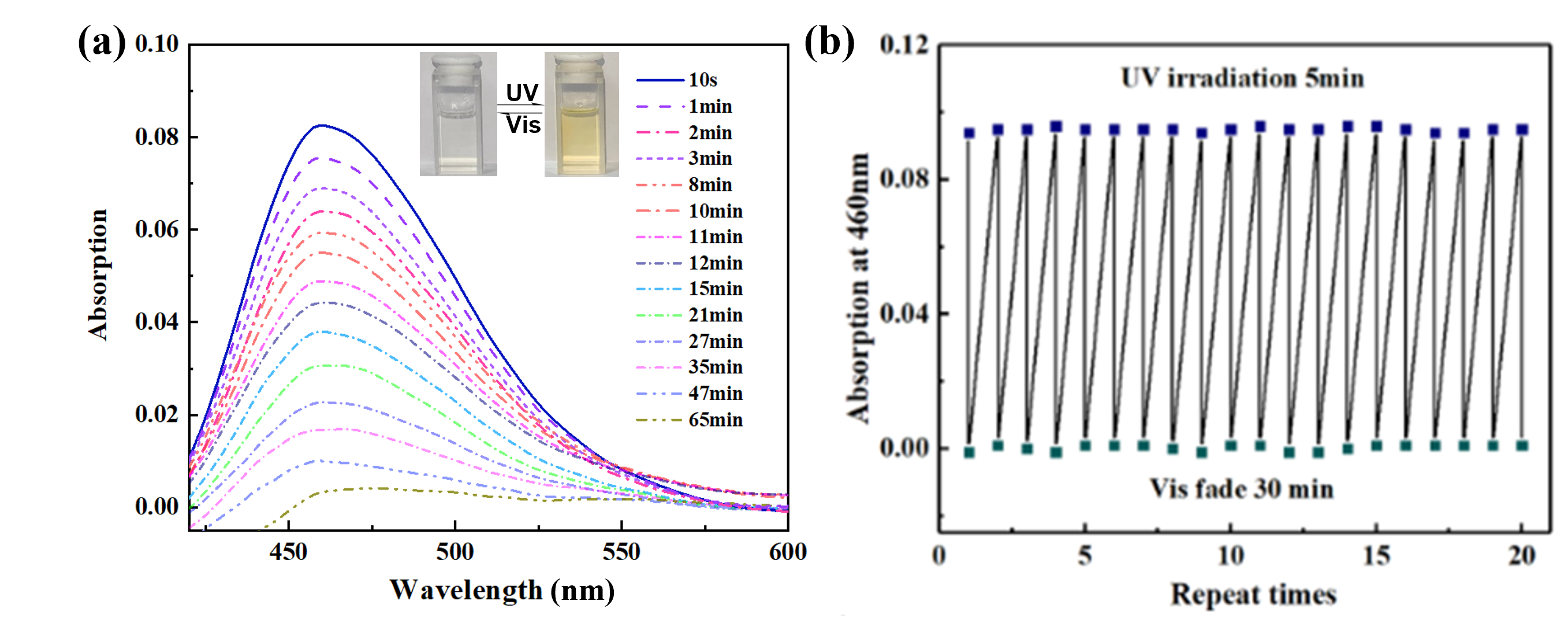


**Figure S1**. (a) UV-Vis absorption spectra of TrPE_2_-MA in tetrahydrofuran solution (Inset: Discoloration diagram of TrPE_2_-MA in 0.1 mol/L tetrahydrofuran solution). (b) Recycling of the photochromic processes for TrPE_2_-MA in solution state as a function of exposure to UV light (365 nm) and visible light for 5 minutes and 30 minutes, respectively.


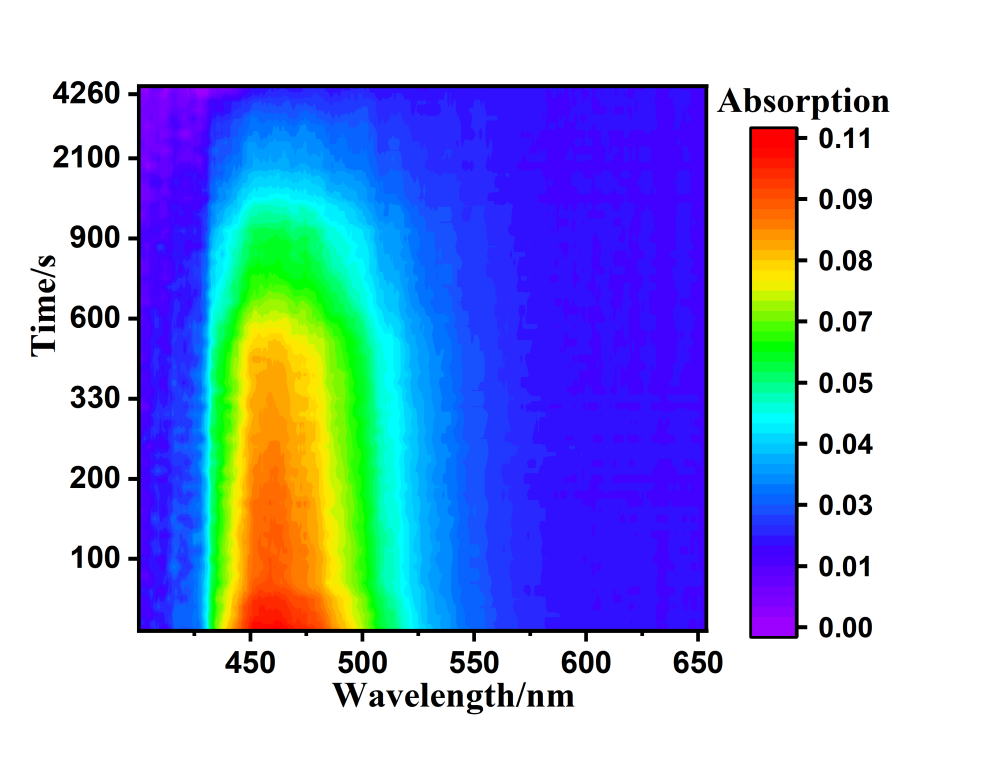


**Figure S2.** Time-dependent UV-Vis absorption spectra of the printed film based on Tr SMP under UV-light (365 nm) and white light.


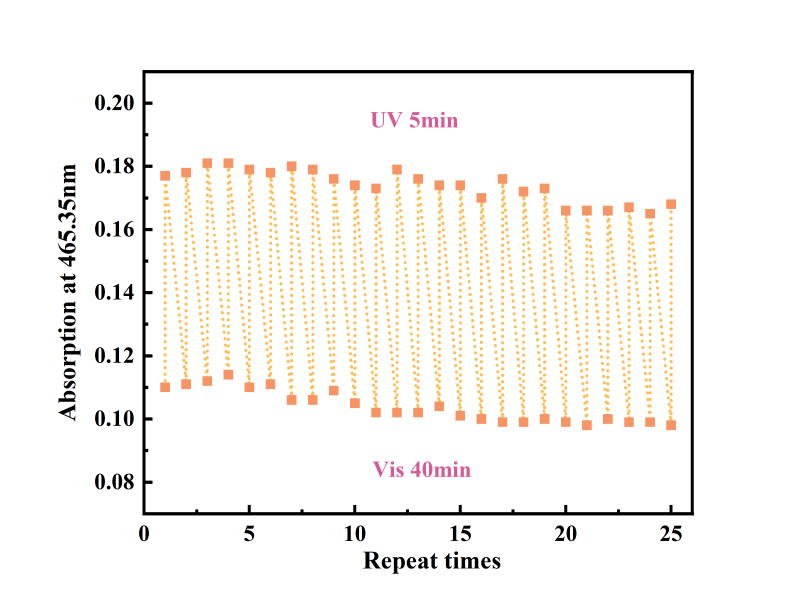


**Figure S3.** Cyclicity test of the printed film based on Tr SMP under UV-light (365 nm) and white light.


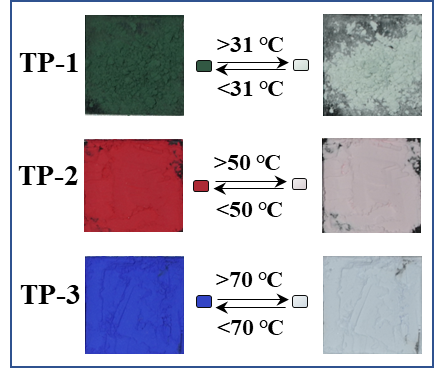


**Figure S4.** Schematic diagram of color change at different temperature nodes of TP-1, TP-2 and TP-3.


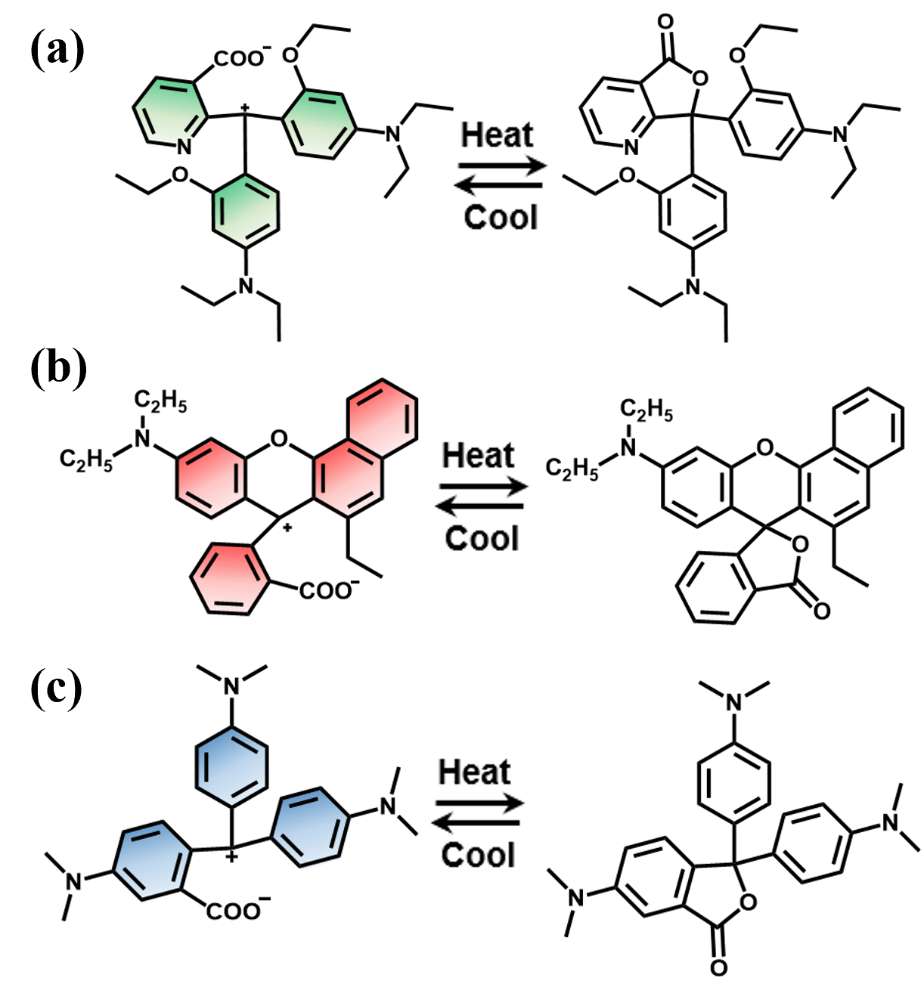


**Figure S5.** Diagrams of the molecular structures and color-changing mechanisms of molecular structures of three thermochromic pigments (a: TP-1, b: TP-2, c: TP-3).


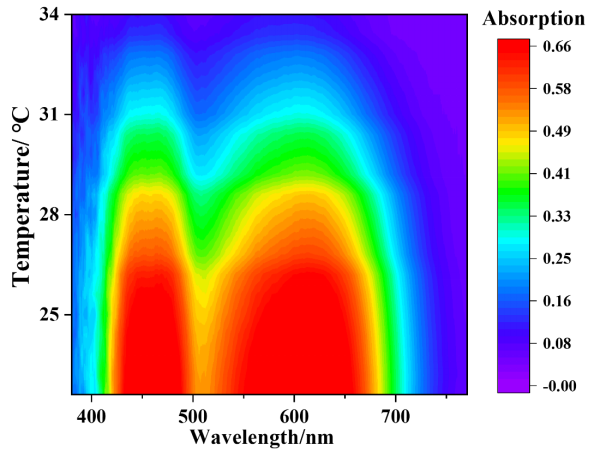


**Figure S6.** Time-dependent UV-Vis absorption spectra of TP-1 under different temperatures.


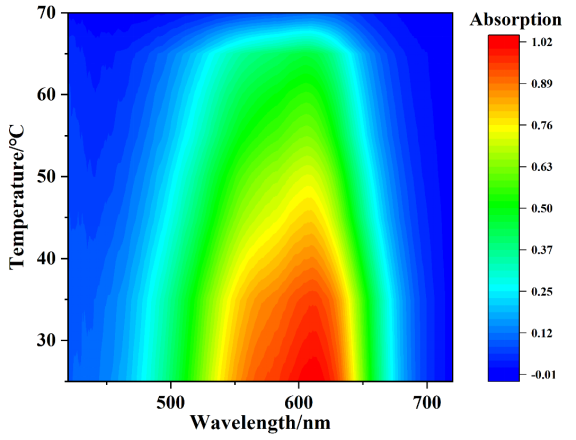


**Figure S7.** Time-dependent UV-Vis absorption spectra of TP-2 under different temperatures.


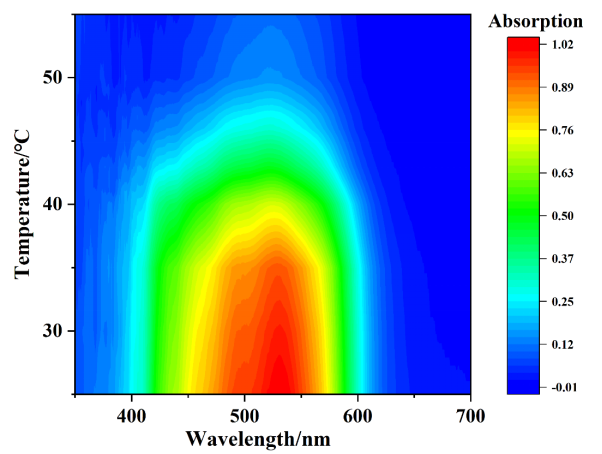


**Figure S8.** Time-dependent UV-Vis absorption spectra of TP-3 under different temperatures.


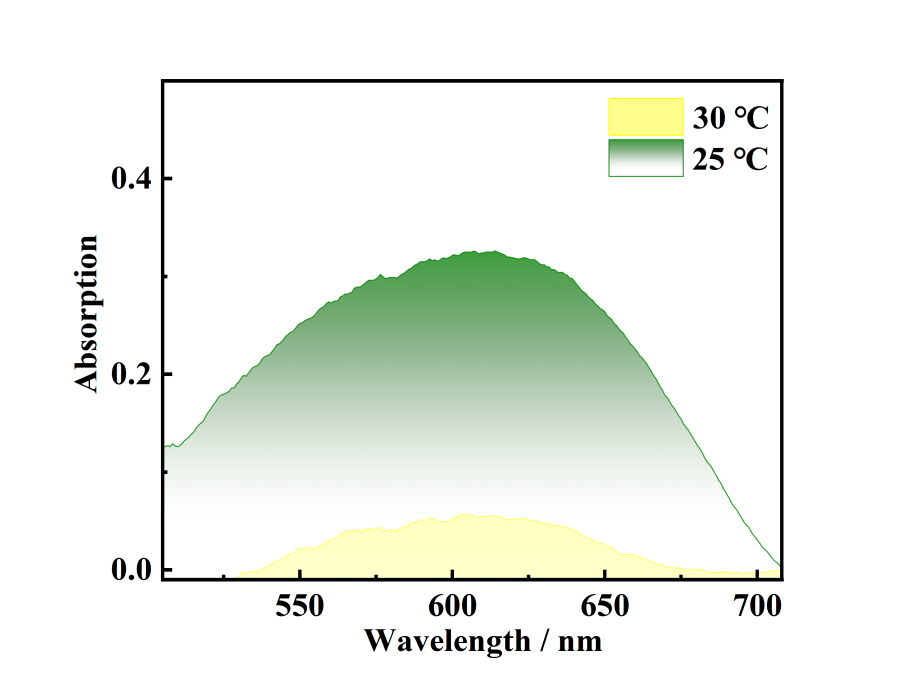


**Figure S9**. UV-Vis absorption spectrum of temperature variation based on TP1 material.


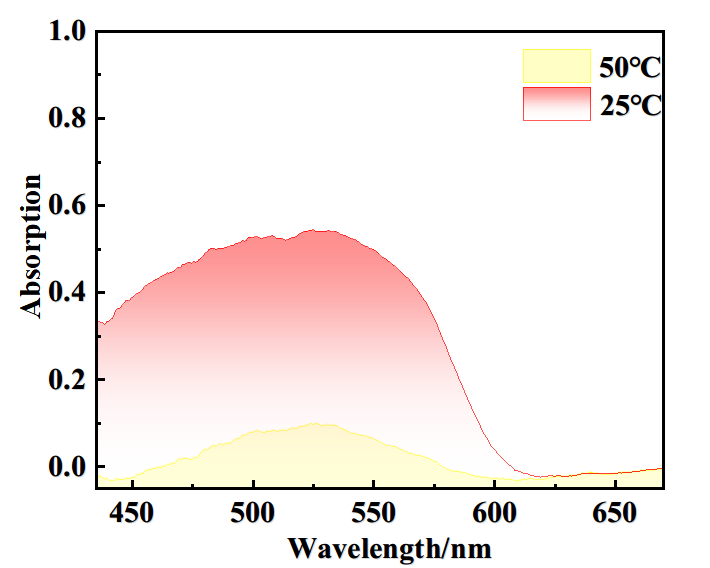


**Figure S10.** UV-Vis absorption spectra of printed structure with TP-2 material.


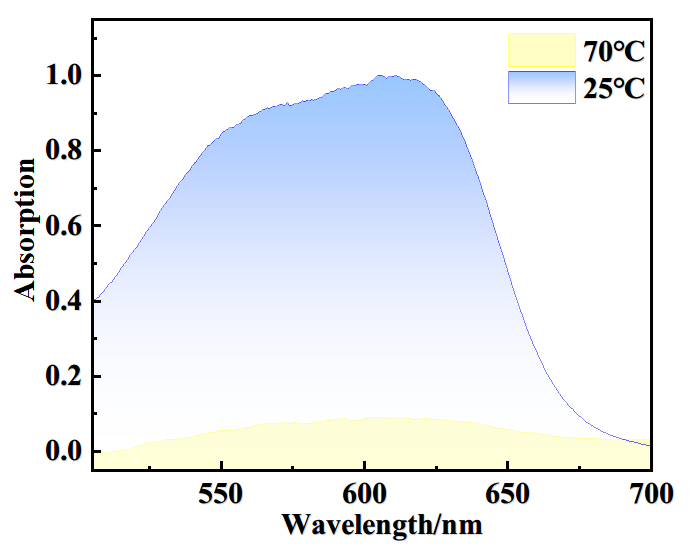


**Figure S11.** UV-Vis absorption spectra of printed structure with TP-3 material.


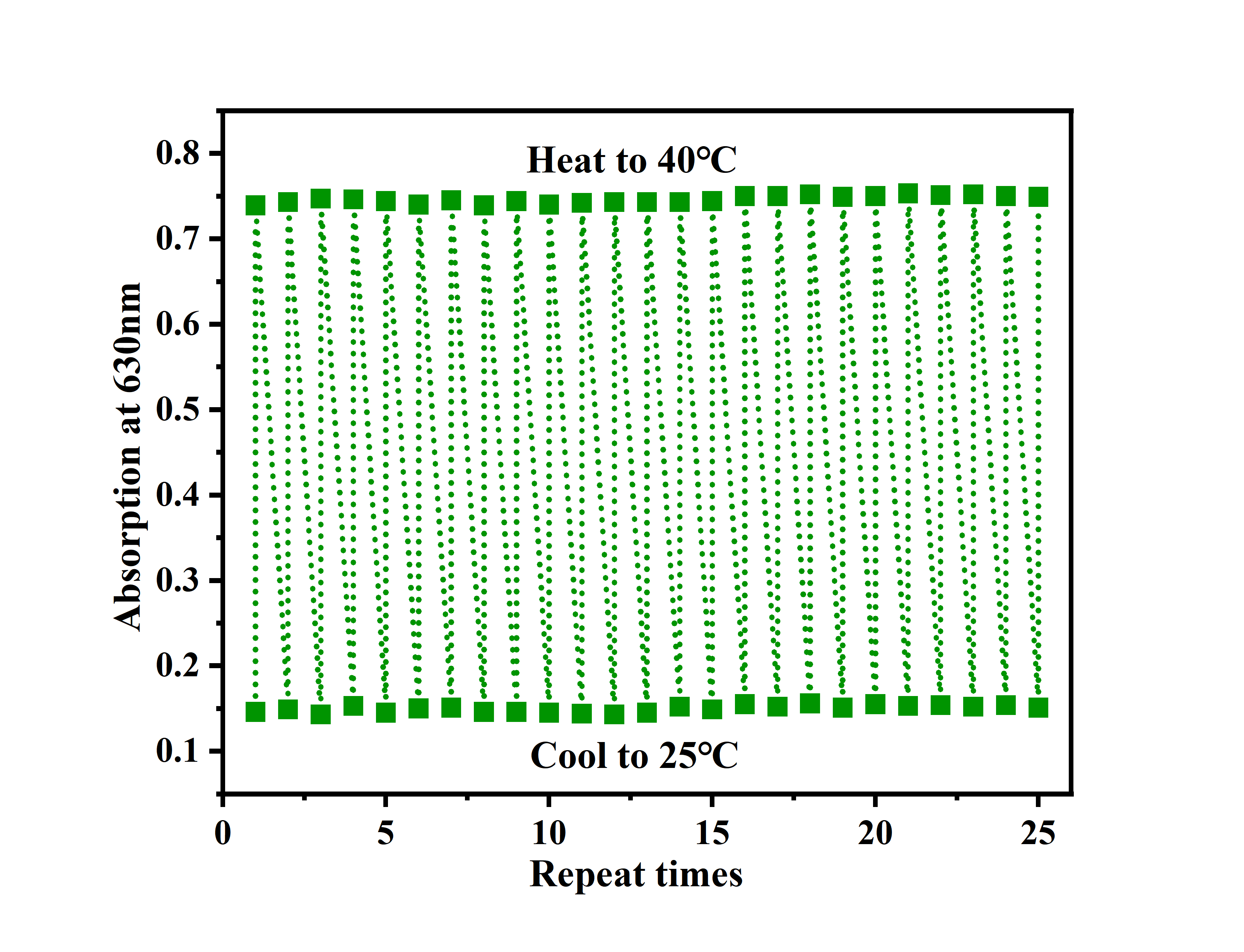


**Figure S12.** Cycle test of temperature variation based on TP1 material.


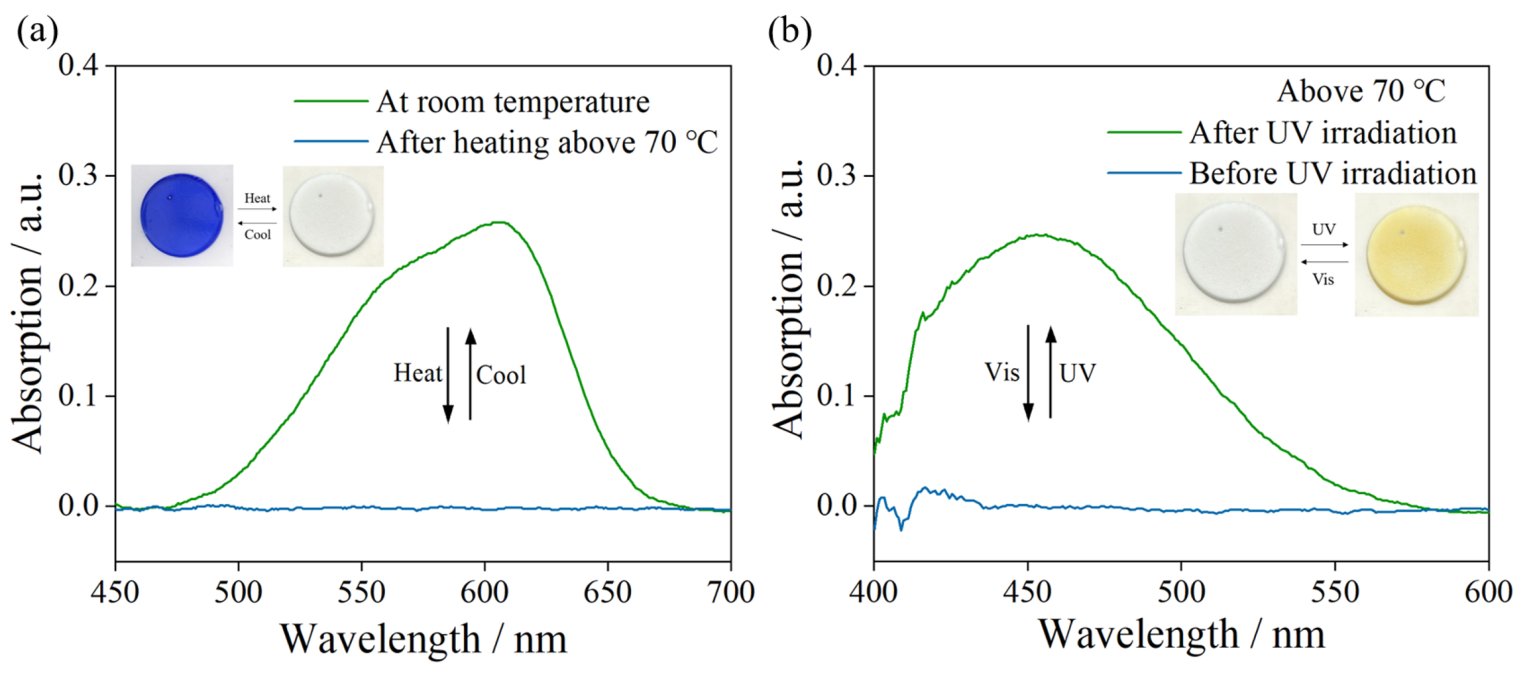


**Figure S13.** UV-vis absorption spectra of Tr-TP3 PMMA film, (a) at room temperature and above 70 ℃; (b) before and after UV irradiation above 70 ℃.


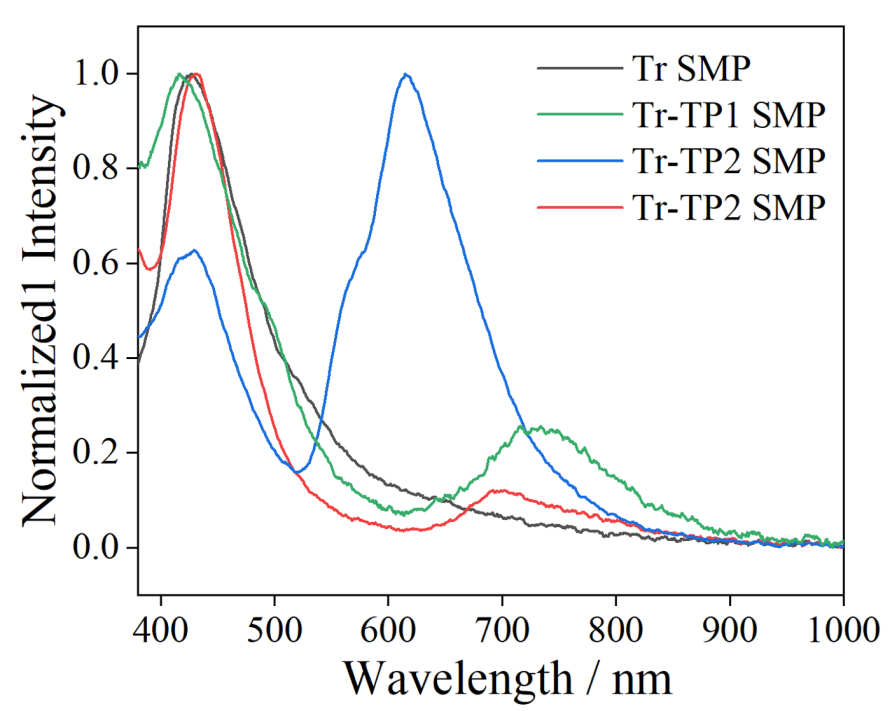


**Figure S14.** Normalized emission spectrum of Tr-TP1, Tr-TP2, Tr-TP3, and Tr SMP under excitation at 310 nm and at room temperature.


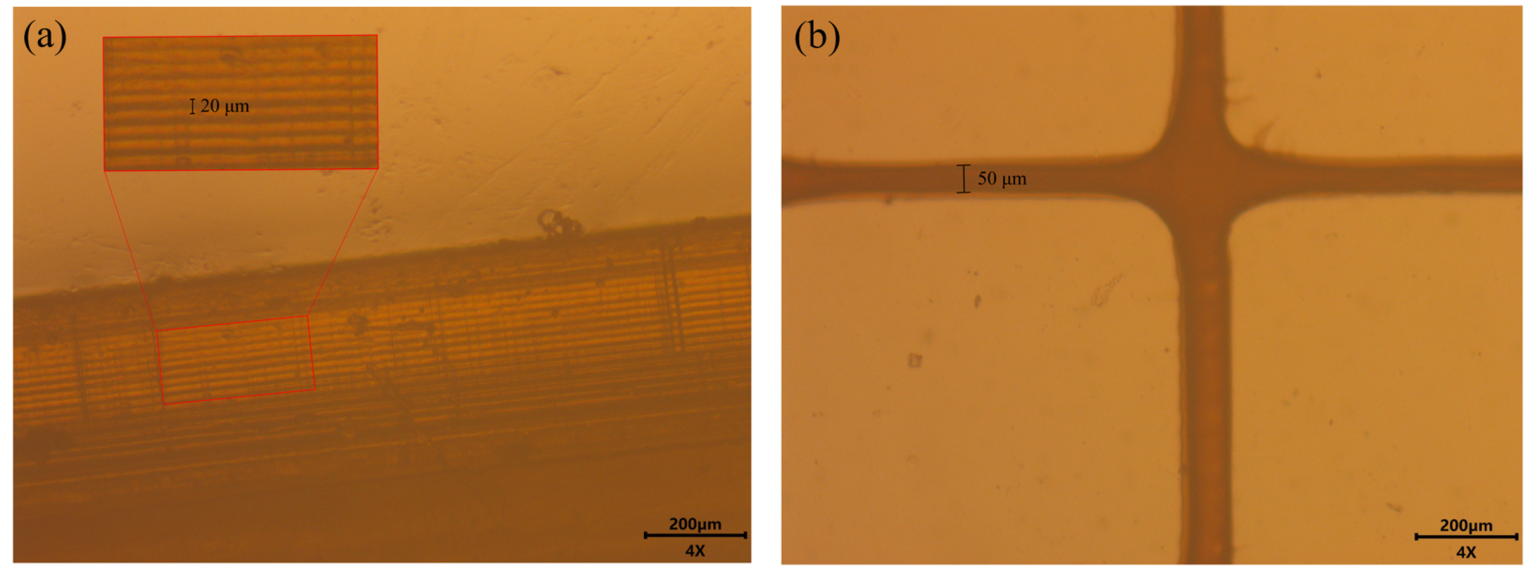


**Figure S15.** Optical microscope images of the 3D printed structures. (a) vertical plane, (b) horizontal plane.


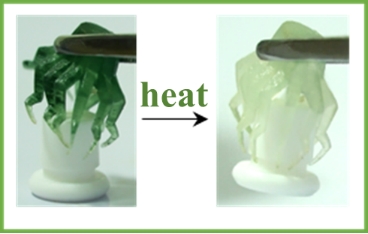


**Figure S16.** Schematic diagram of gripper pinching object while changing color after shape memory recovery.


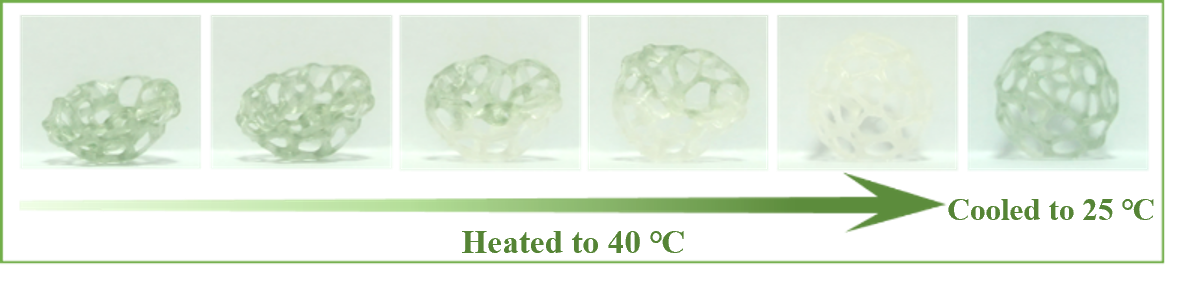


**Figure S17.** Shape memory recovery diagram of 3D printed buckliball.


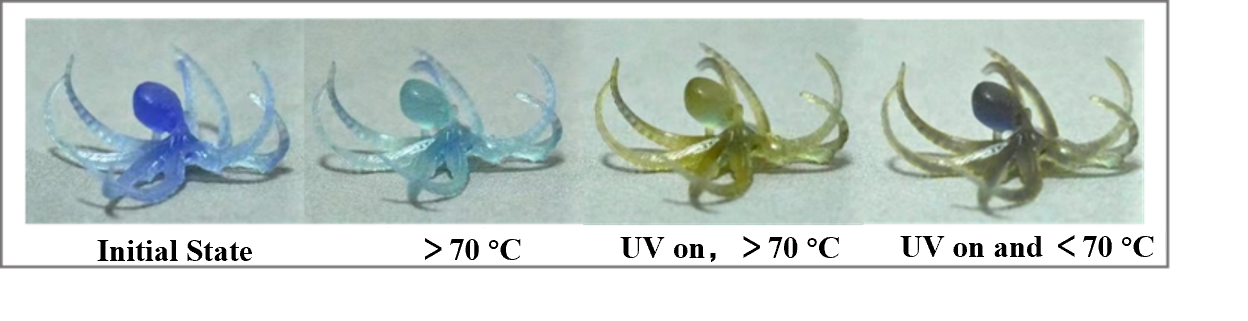


**Figure S18.** Colour-changing effect diagram of 3D printed octopus.


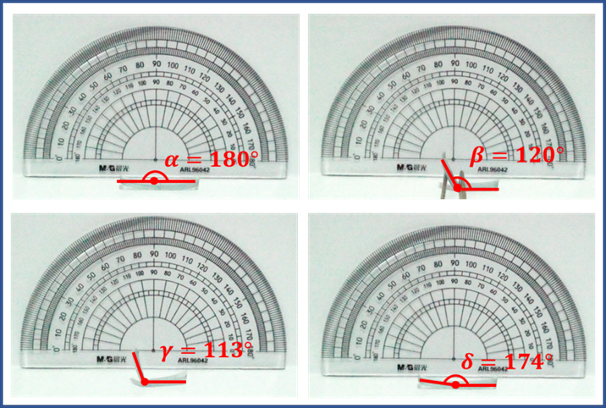


**Figure S19.** The calculation process of shape memory fixation rate and shape memory recovery rate.


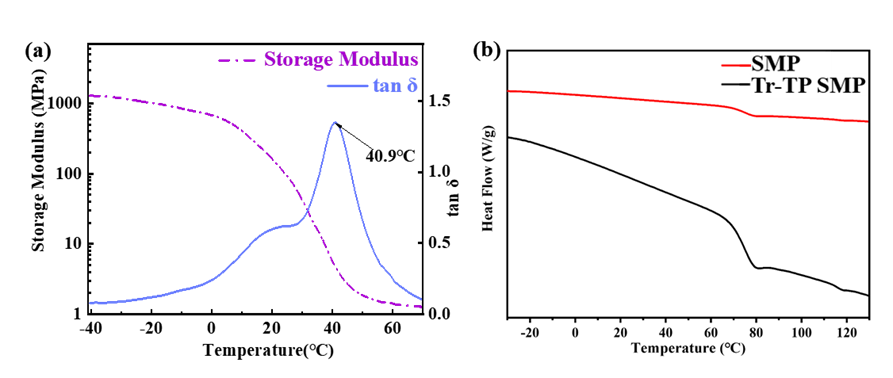


**Figure S20.** (a) Dynamic mechanical analysis (DMA) of printed structures based on SMP. (b) DSC curves of solidified polymer with different liquid resins after polymerization.


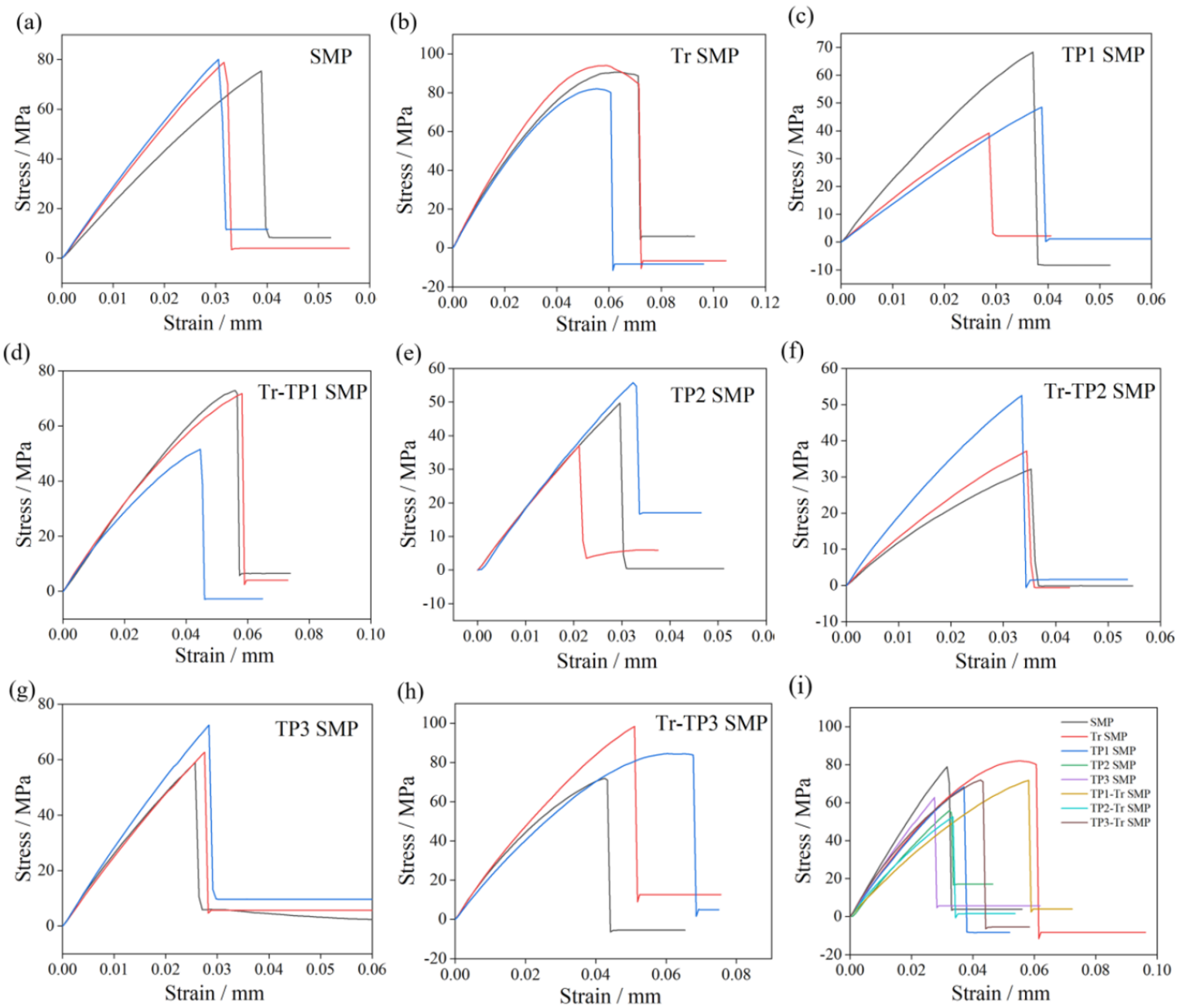


**Figure S21**. Mechanical tensile curves of SMP (a), Tr SMP (b), TP1 SMP (c), Tr-TP1 SMP (d), TP2 SMP (e), Tr-TP2 SMP (f), TP3 SMP (g), Tr-TP3 SMP (h), and all SMPs (i) at room temperature and visible light.


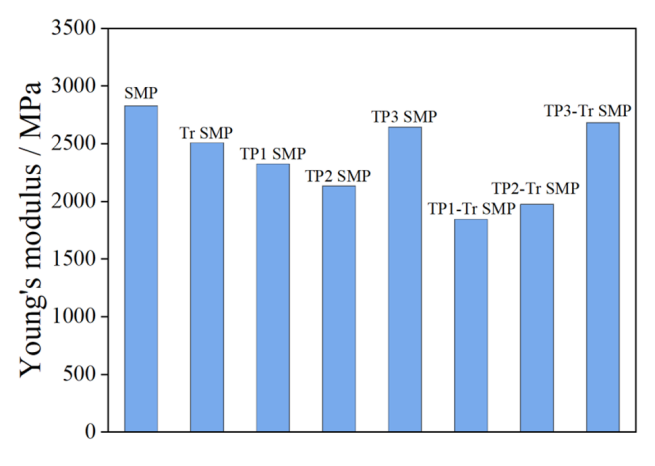


**Figure S22.** Young’s Modulus of all SMPs at room temperature and visible light.


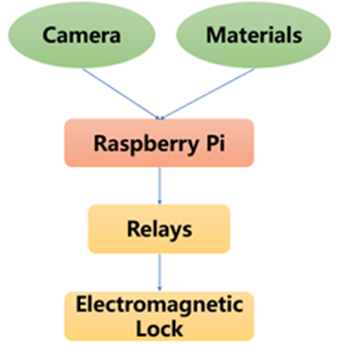


**Figure S23.** The hardware composition in the encryption process.

Supplementary Tables

**Table S1.** Materials used in the color-changing liquid resin.

| **Structure** | **Image** | **Intensity (normalization)** | **Intensity**  **(Mw/cm^2^)** | **T_1_^a)^**  **(s)** | **Thinness**  **(μm)** | **T_2_^b)^**  **(s)** |
| --- | --- | --- | --- | --- | --- | --- |
| Snowflake | 1 | 120 | 12.7 | 1.2 | 25 | 0.0 |
|  | 19 | 120 | 12.7 | 1.2 |  | 2.0 |
|  | 40 | 120 | 12.7 | 1.15 |  | 2.0 |
| Eiffel Tower | 1 | 130 | 13.6 | 1.2 | 50 | 0.0 |
|  | 54 | 130 | 13.6 | 1.2 |  | 1.5 |
|  | 150 | 125 | 13.2 | 1.08 |  | 1.5 |
|  | 200 | 120 | 12.7 | 1.08 |  | 1.5 |
| Heart-Box | 1 | 110 | 11.8 | 0.8 | 50 | 0.0 |
|  | 12 | 110 | 11.8 | 0.8 |  | 1.5 |
|  | 66 | 115 | 12.3 | 0.8 |  | 1.0 |
| Octopus | 1 | 110 | 11.8 | 1 | 50 | 0.0 |
|  | 49 | 110 | 11.8 | 0.85 |  | 1.0 |
|  | 150 | 110 | 11.8 | 0.8 |  | 1.0 |
|  | 100 | 110 | 11.8 | 0.55 |  | 0.8 |
| Buckliball | 1 | 110 | 11.8 | 0.8 | 50 | 0.0 |
|  | 295 | 115 | 12.3 | 0.8 |  | 1 |

a) T_1_: Curing time for each layer, b) T_2_: Waiting time for each layer

**Table S2.** The materials used to 3D print the structure

| **Structure** | **Snowflake** | **Eiffel Tower** | **Heart-Box** | **Octopus** | **Buckliball** |
| --- | --- | --- | --- | --- | --- |
| Materials | Tr-TP3 SMP | Tr-TP3 SMP | Tr-TP2 SMP /Heart  TP1 SMP /Foldable wings | Tr-TP3 SMP | TP1 SMP |

Supplementary Movies

**Video S1.** Shape Change of Spline

**Video S2.** Shape Memory Change and Color Change of Printed Gripper

**Video S3.** Shape Memory Change of Printed Eiffel Tower

**Video S4.** Deformation and discoloration process
